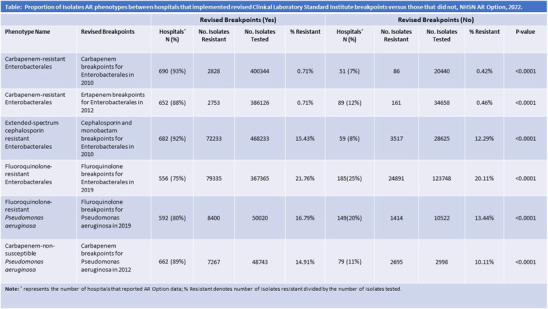# Uptake of Revised CLSI Breakpoints and Potential Impact among Hospitals Reporting to The NHSN AR Option, 2022

**DOI:** 10.1017/ash.2024.167

**Published:** 2024-09-16

**Authors:** Allan Nkwata, Rupert England, Laura Blum, Hsiu Wu

**Affiliations:** Centers for Disease Control and Prevention; Lantana Consulting Group Contractor for CDC

## Abstract

**Background:** Antimicrobial susceptibility testing (AST) is critical for detecting antimicrobial resistance (AR) and guiding antimicrobial treatment. The Clinical and Laboratory Standards Institute (CLSI) regularly publishes and revises breakpoints to guide the interpretation of AST results. In 2010–2019, CLSI has lowered many breakpoints for Enterobacterales and Pseudomonas aeruginosa. Timely implementation of updated breakpoints can vary across hospital laboratories, leading to shifts in the interpretation of AST results. This issue is a potential threat to the estimation of national prevalence estimates for AR and limits the comparability of AR data across hospitals. Hospitals submit AST data with clinical laboratory interpretations to the AR Option of CDC’s National Healthcare Safety Network (NHSN). NHSN tracks whether a hospital adopted the revised CLSI breakpoints for six organism-antimicrobial combinations involving AR phenotypes commonly associated with healthcare associated infections through hospital self-reporting status into a structured survey — 2022 NHSN Annual Hospital Survey (Table). For this analysis, we describe the uptake of revised CLSI breakpoints and compare cumulative antibiograms among hospitals that used various breakpoints. **Methods:** We included hospitals that completed the 2022 NHSN annual survey and submitted data to the NHSN AR Option for at least 9 months in 2022 by November 1, 2023. The percentage of hospitals that implemented CLSI breakpoints, published during 2010–2019, were determined for combinations of antibiotic class, organism, and CLSI revision year (Table). We calculated percent resistance (%R) as the number of isolates meeting AR phenotype definitions divided by the total number of Isolates tested for the following phenotypes: carbapenem-resistant Enterobacterales (CRE) which included E. coli, Klebsiella, and Enterobacter species; extended-spectrum cephalosporin-resistant Enterobacterales (ESC); carbapenem-non-susceptible P. aeruginosa; fluoroquinolone-resistant P. aeruginosa; and fluoroquinolone-resistant Enterobacterales. **Results:** Among the 741 hospitals included, 75%–93% implemented any of the six revised CLSI breakpoints (Table). The %R was higher among isolates from hospitals that adopted revised breakpoints compared to those that did not (p < 0 .0001). The largest difference was observed for carbapenem-non-susceptible P. aeruginosa (14.91% vs 10.11%). **Conclusions:** The uptake of revised CLSI breakpoints varied across hospitals, organism-antimicrobial combinations, and CLSI versions. This analysis indicates that the prevalence of AR for corresponding phenotypes could be underestimated if data from hospitals using higher, outdated breakpoints are included. It is important for NHSN to continue tracking breakpoints used in individual hospitals and encourage hospitals to report complete data on the original AST results, such as MIC, to optimize the accuracy of national AR surveillance.

**Figure:**